# Diaphragmatic excursion is correlated with the improvement in exercise tolerance after pulmonary rehabilitation in patients with chronic obstructive pulmonary disease

**DOI:** 10.1186/s12931-021-01870-1

**Published:** 2021-10-22

**Authors:** Masashi Shiraishi, Yuji Higashimoto, Ryuji Sugiya, Hiroki Mizusawa, Yu Takeda, Shuhei Fujita, Osamu Nishiyama, Shintarou Kudo, Tamotsu Kimura, Yasutaka Chiba, Kanji Fukuda, Yuji Tohda, Hisako Matsumoto

**Affiliations:** 1 Department of Rehabilitation Medicine, Kindai University School of Medicine, 377-2 Onohigashi, Osakasayama, Osaka 5898511 Japan; 2 Department of Respiratory Medicine and Allergology, Kindai University School of Medicine, Osaka, Japan; 3 Inclusive Medical Science Research Institute, Morinomiya University of Medical Sciences, Osaka, Japan; 4 Division of Biostatistics, Clinical Research Center, Kindai University School of Medicine, Osaka, Japan

**Keywords:** Pulmonary rehabilitation, Diaphragmatic excursion, COPD, Six-minute walk distance (6MWD)

## Abstract

**Background:**

In patients with chronic obstructive pulmonary disease (COPD), the maximum level of diaphragm excursion (DE_max_) is correlated with dynamic lung hyperinflation and exercise tolerance. This study aimed to elucidate the utility of DE_max_ to predict the improvement in exercise tolerance after pulmonary rehabilitation (PR) in patients with COPD.

**Methods:**

This was a prospective cohort study. Of the 62 patients with stable COPD who participated in the outpatient PR programme from April 2018 to February 2021, 50 completed the programme. Six-minute walk distance (6MWD) was performed to evaluate exercise tolerance, and ultrasonography was performed to measure DE_max_. Responders to PR in exercise capacity were defined as patients who demonstrated an increase of > 30 m in 6MWD. The receiver operating characteristic (ROC) curve was used to determine the cut-off point of DE_max_ to predict responses to PR.

**Results:**

Baseline levels of forced expiratory volume in 1 s, 6MWD, maximum inspiratory pressure, DE_max_ and quadriceps muscle strength were significantly higher, and peak dyspnoea of modified Borg (mBorg) scale score was lower in responders (n = 30) than in non-responders (n = 20) to PR (p < 0.01). In multivariate analysis, DE_max_ was significantly correlated with an increase of > 30 m in 6MWD. The area under the ROC curve of DE_max_ to predict responders was 0.915, with a sensitivity and specificity of 83% and 95%, respectively, at a cut-off value of 44.9 mm of DE_max_.

**Conclusion:**

DE_max_ could adequately predict the improvement in exercise tolerance after PR in patients with COPD.

## Background

Chronic obstructive pulmonary disease (COPD) is a progressive disease characterised by minimally reversible airflow limitation [1]. The main feature of COPD is the inability of patients to cope with their activities of daily life due to shortness of breath. Although the pathophysiological mechanisms involved in the development of dyspnoea and poor exercise tolerance in patients with COPD are complex, dynamic lung hyperinflation (DLH) plays a central role [2] by increasing ventilatory workload and decreasing the pressure-generating capacity of the inspiratory muscles.

Pulmonary rehabilitation (PR) is a non-pharmacological intervention and has been reported to improve dyspnoea, exercise capacity and quality of life of patients with COPD [3]. Owing to a body of evidence, PR is now established as the standard of care for patients with COPD [4]. However, not all patients with COPD benefit from PR to the same extent. Therefore, identifying patients who are likely to achieve maximum benefit from the PR programme is crucial. So far, several studies have shown that severe airflow limitation or poor exercise tolerance at baseline may predict a better response to PR [5, 6], but another study has reported inconsistent findings [7]. Furthermore, one study reported that patients with severe dyspnoea did not respond well to PR and patients with milder dyspnoea responded well [8].

Considering the role of DLH in the development of dyspnoea and poor exercise tolerance in patients with COPD, objective measures that reflect the degree of DLH may help in identifying good responders to PR. Previously, we reported that there was an association between increased dyspnoea due to DLH on exercise and decreased exercise capacity in patients with COPD and reduced mobility of the diaphragm, which was assessed by the maximum level of diaphragm excursion (DE_max_) using ultrasonography [9]. Other research groups reported the utility of ultrasonographic assessment of diaphragmatic mobility in COPD in understanding its association with 6-min walk distance (6MWD), dyspnoea [10] and increased mortality [11].

However, there have been no reports on the association between diaphragmatic mobility and the effect of PR to improve exercise tolerance. The primary aim of this study is to clarify the role of DE_max_ to predict the improvement in exercise tolerance after PR in patients with COPD.

## Materials and methods

### Study design and subjects

This was a single-centre, observational, prospective cohort study. The study included 62 patients with clinically stable COPD who visited the Department of Respiratory Medicine and Allergology, Kindai University Hospital, between April 2018 and February 2021. The exclusion criteria included unstable medical conditions that could cause or contribute to breathlessness, such as metabolic, cardiovascular or other respiratory diseases, or any other disorders that could interfere with exercise testing, such as neuromuscular diseases or musculoskeletal problems. This study was approved by the Ethics Committee of Kindai University School of Medicine. Written informed consent was obtained from all participants.

### Measurements

**Fig. 1:**
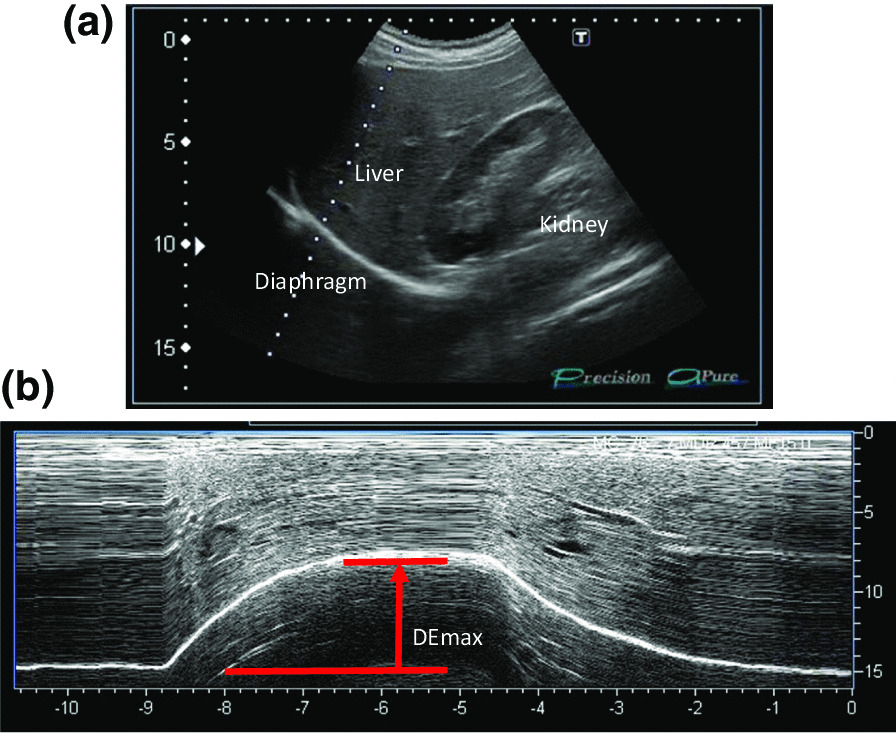
Representative image of the right diaphragm. The probe was positioned below the right costal margin between the midclavicular and anterior axillary lines. **A** Two-dimensional ultrasonographic image of the right hemidiaphragm (B-mode). Diaphragmatic movements were recorded in M-mode during deep breathing (DE_max_) (**B**)

All participants underwent ultrasonography (Xario 200, Toshiba, Tokyo, Japan) for the assessment of their DE_max_. Using the liver as an acoustic window (Fig. 1A), a convex 3.5 MHz probe was used to measure the excursions of the right hemidiaphragm according to the techniques mentioned in previous studies [9, 12, 13]. The M-mode cursor was rotated and placed on the axis of diaphragmatic displacement on the stored image, and displacement measurements were performed. Measurements were performed during each of the three deep breaths, and DE_max_ was measured (Fig. 1B). The maximum value obtained for the three deep breaths was used. 6MWD was performed to evaluate walking capacity according to the American Thoracic Society (ATS)/European Respiratory Society (ERS) statement [14–16]. All participants performed the 6MWD test before and after the PR programme, and the magnitude of their perceived breathlessness and their leg fatigue was rated using a 1–10-point Borg scale. Responders to PR in exercise capacity were defined as those who demonstrated more than 30 m increase in 6MWD after the PR programme, which was the definition of minimal clinically important difference (MCID) for 6MWD [17].

Spirometry (CHESTAC-800, Chest, Tokyo, Japan) was performed following the 2005 ATS/ERS recommendations [18] for measuring forced vital capacity (FVC), forced expiratory volume in 1 s (FEV_1_) and inspiratory capacity. Respiratory muscle strength was assessed by measuring the maximum inspiratory pressure (PI_max_) generated against an occluded airway at residual volume [19] (SP-370, Fukuda Denshi, Tokyo, Japan). A hand-held dynamometer (μTasF-1, Anima Corp., Tokyo) was used to measure quadriceps muscle strength (QMS). The impact of COPD on health status was assessed using the COPD assessment test (CAT), a patient-completed questionnaire on eight items, namely, cough, phlegm, chest tightness, breathlessness, limited activities, confidence leaving home, sleeplessness and energy. The scores for each of the items range from 0 to 5 points, resulting in a CAT total score ranging from 0 to 40 points [20], and MCID of CAT is 2 points [21]. In all patients with COPD, emphysema was evaluated by computed tomography of the chest. A SYNAPSE VINCENT volume analyser (FUJIFILM Medical, Tokyo, Japan) was used to measure the low attenuation area (%LAA).

### Rehabilitation programme

The outpatient PR programme was conducted twice a week for 12 weeks (24 sessions), including aerobic exercise training (ergometer and walking exercise) at 60–70% of peak workload for 20–40 min and upper- and lower-limb muscle strength training for 10–20 min.

### Sample size

The sample size was estimated using R software. The analysis based on 6MWD data from the PR programme revealed that 40 subjects were required if the expected area under the curve (AUC) below the receiver operating characteristic (ROC) curve was 0.80, the power was 90%, and the significance level was 0.01. Furthermore, we anticipated a dropout from the PR programme. Thus, we set the sample size to 50 participants.

### Statistical analysis

Responders and non-responders were compared using *t*-test, the Wilcoxon rank-sum test or χ^2^ test, as appropriate. The paired *t*-test or the Wilcoxon signed-rank test was used to evaluate the changes in the parameters before and after the PR programme. The Pearson correlation coefficient was used to analyse the relationship between changes in 6MWD and independent variables because changes in 6MWD were normally distributed. Additionally, multivariate logistic regression models were used to assess the ability of variables to predict a response to PR. The ROC curve method was used to assess the ability of DE_max_ to predict a response to PR. All statistical analyses were performed using the JMP software programme (JMP®, Version 14; SAS Institute Inc., Cary, NC, USA).

## Result

**Fig. 2:**
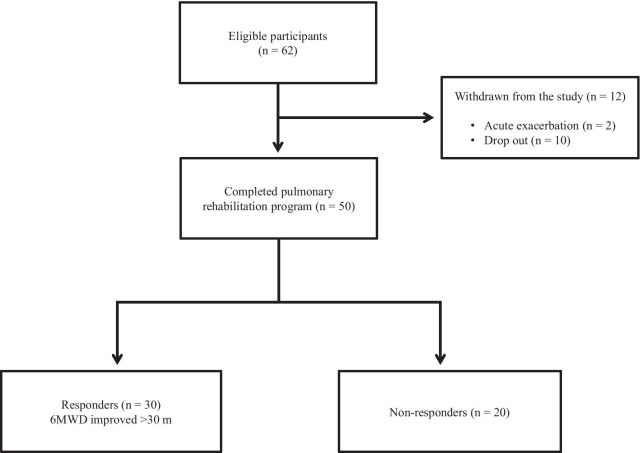
Study flow diagram. *COPD* chronic obstructive pulmonary disease, *PR* pulmonary rehabilitation, *6MWD* 6-min walk distance

**Fig. 3:**
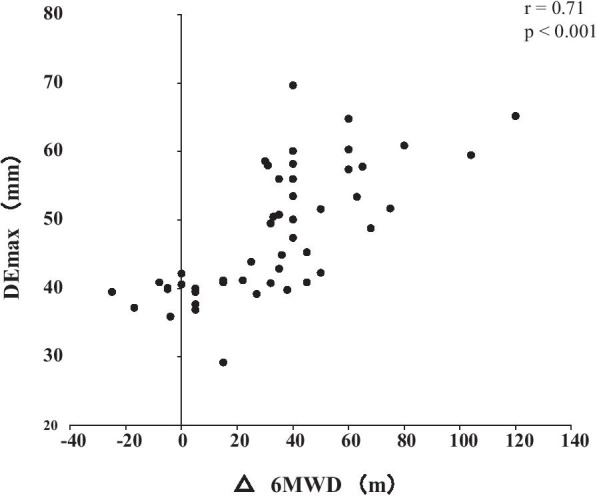
Relationship between DE_max_ and the changes in 6MWD after pulmonary rehabilitation. Changes in 6MWD were significantly positively correlated with DE_max_ (r = 0.72; p < 0.001). *DE*_*max*_ maximum diaphragmatic excursion, *6MWD* 6-min walk distance

Out of the 62 patients included in the study, 50 completed the PR programme (Fig. 2). Two patients dropped out because of severe exacerbation of COPD, and 10 patients discontinued the PR owing to the coronavirus pandemic. Table 1 presents the baseline characteristics of the participants. After the PR programme, scores for CAT, 6MWD, peak dyspnoea and leg fatigue of the modified Borg (mBorg) scale, and QMS improved significantly (Table 2). Thirty patients showed an increase of > 30 m in 6MWD after PR (responders: 60%), and 20 patients (40%) were defined as non-responders. Baseline levels of %FEV_1_, 6MWD, PI_max_, DE_max_ and QMS were significantly higher and those of CAT score and peak dyspnoea of mBorg scale were significantly lower in responders than in non-responders (Table 1). Changes in 6MWD were significantly correlated with baseline levels of CAT, %FEV_1_, peak dyspnoea of mBorg scale, PI_max_, DE_max_ (Fig. 3) and QMS and marginally correlated with baseline levels of 6MWD (Table 3).

**Table 1:** Baseline characteristics of study participants

	All	Responders	Non-responders	p value
	n = 50	n = 30	n = 20	
Age, year	74.9 ± 5.7	74.6 ± 5.8	75.3 ± 5.7	0.95
Gender, male/female (%)	44/6 (88/12)	28/2 (93/7)	16/4 (81/19)	0.16
BMI, kg/m^2^	22.1 ± 3.6	22.0 ± 2.8	22.3 ± 4.7	0.80
GOLD (I + II/III/IV)	27/17/6	20/8/2	7/9/4	0.07
LTOT, n (%)	5 (10)	3 (10)	2 (10)	0.89
CAT	14 (10–20)	11.5 (8.8–16.8)	18.5 (12.3–20.8)	0.04
FVC %predicted	93.0 ± 20.2	97.3 ± 19.5	86.4 ± 20.0	0.06
FEV_1_ %predicted	56.0 ± 21.8	63.2 ± 21.8	45.1 ± 17.1	0.003
SpO_2_, %	90 ± 7	91 ± 7	90 ± 6	0.55
%LAA	17.5 ± 16.3	17.0 ± 14.6	18.2 ± 19.0	0.65
6MWD, m	378 ± 88	411 ± 83	328 ± 71	< 0.001
mBorg scale dyspnoea	5 (4–7)	4 (3–7)	6 (4–8)	0.007
mBorg scale leg fatigue	2 (1–5)	2 (0.9–4)	2.5 (1.3–5)	0.50
PI_max_, cmH_2_O	55.7 ± 21.9	65.7 ± 19.4	40.6 ± 16.2	< 0.001
DE_max_, mm	47.9 ± 9.3	52.9 ± 7.8	40.4 ± 5.3	< 0.001
QMS, kgf/kg	0.54 ± 0.14	0.58 ± 0.13	0.48 ± 0.13	0.007

*COPD* chronic obstructive pulmonary disease, *BMI* body mass index, *GOLD* Global Initiative for Chronic Obstructive Lung Disease, *LTOT* long-term oxygen therapy, *CAT* COPD assessment test, *FVC* forced vital capacity, *FEV*_*1*_ forced expiratory volume in 1 s, *SpO*_*2*_ saturation of percutaneous oxygen, *LAA* low attenuation area, *6MWD* 6-min walk distance, *mBorg* modified Borg, *PI*_*max*_ maximum inspiratory pressure, *DE*_*max*_ maximum diaphragmatic excursion, *QMS* quadriceps muscle strength. Values are presented as means ± standard deviations or median (inter-quartile)

**Table 2:** Effects of pulmonary rehabilitation (n = 50)

	Baseline	After PR	p value
CAT	14 (10–20)	10 (5.8–18)	< 0.001
6MWD, m	378 ± 88	411 ± 100	< 0.001
mBorg scale dyspnoea	5 (4–7)	4 (2–6)	0.02
mBorg scale leg fatigue	2 (1–5)	2 (0.9–3)	< 0.001
QMS, kgf/kg	0.54 ± 0.14	0.72 ± 0.13	< 0.001

*CAT* COPD assessment test, *6MWD* 6-min walk distance, *mBorg* modified Borg, *QMS* quadriceps muscle strength. Values are presented as means ± standard deviations or median (inter-quartile)

**Table 3:** Correlations between changes in 6MWD with diaphragm excursion and baseline characteristics

Independent variable	Total patients (n = 50)	
	Pearson correlation	p value
	coefficient (r)	
Age, year	− 0.10	0.48
BMI, kg/m^2^	− 0.02	0.89
CAT	− 0.34	0.017
FVC %predicted	0.19	0.18
FEV_1_ %predicted	0.45	0.001
6MWD, m	0.28	0.052
mBorg scale dyspnoea	− 0.34	0.017
PI_max_, cmH_2_O	0.58	< 0.001
DE_max_, mm	0.72	< 0.001
QMS, kgf/kg	0.31	0.028

*BMI* body mass index, *CAT* COPD assessment test, *FVC* forced vital capacity, *FEV*_*1*_ forced expiratory volume in 1 s, *6MWD* 6-min walk distance, *mBorg* modified Borg, *PI*_*max*_ maximum inspiratory pressure, *DE*_*max*_ maximum diaphragmatic excursion, *QMS* quadriceps muscle strength

**Fig. 4:**
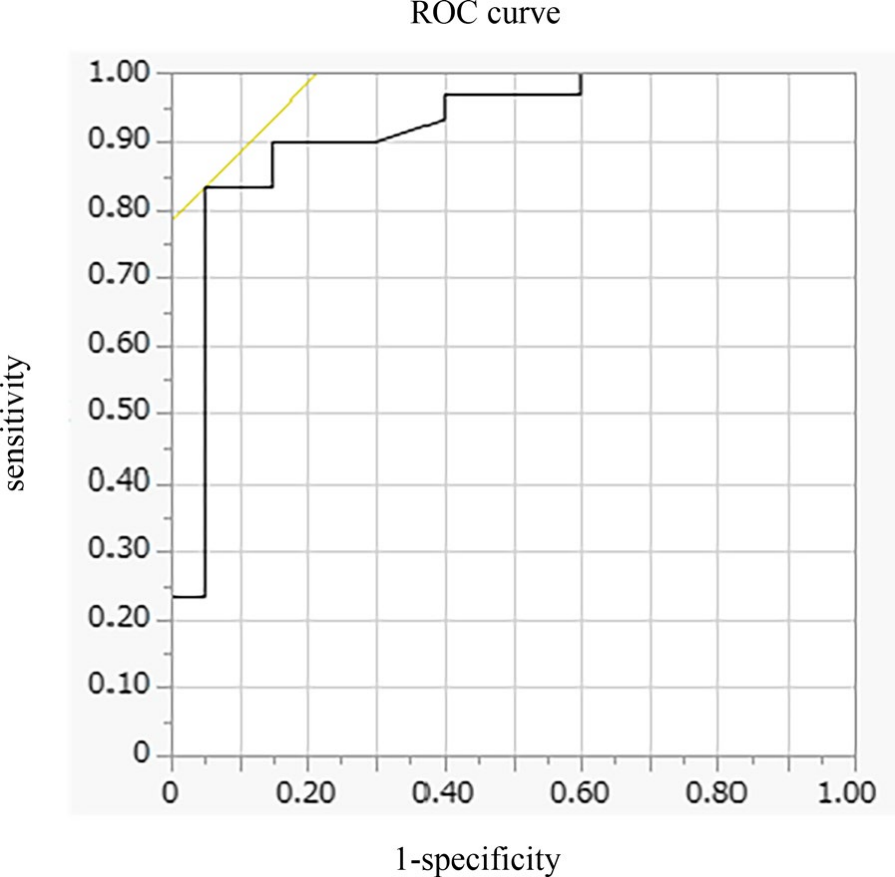
Receiver operating characteristic (ROC) curve for baseline DE_max_ in relation to the response to pulmonary rehabilitation. ROC curve estimates the ability of DE_max_ to predict a clinically important improvement in 6MWD (> 30 m) after pulmonary rehabilitation (AUC = 0.915, sensitivity = 83% and specificity = 95% at a cut-off point of 44.9 mm of DE_max_). *AUC* area under the curve, *6MWD* 6-min walk distance, *DE*_*max*_ maximum diaphragmatic excursion

In multivariate analysis, DE_max_ alone significantly contributed to the prediction of responders (Table 4, Model 1). When using PI_max_ instead of DE_max_ because PI_max_ and DE_max_ showed a strong association (r = 0.73), both PI_max_ and %FEV_1_ contributed to the prediction (Table 4, Model 2). The area under the ROC curve of DE_max_ to predict the responders was 0.915, with a sensitivity of 83% and a specificity of 95% at a cut-off value of 44.9 mm of DE_max_ (Fig. 4). The significance of DE_max_ in the predictability of responders remained even when the analysis was confined to severe patients (%FEV_1_ < 50%, n = 23; AUC = 0.88, sensitivity = 70% and specificity = 100% at a cut-off value of 44.9 mm).

**Table 4:** Multivariate analysis for responders to pulmonary rehabilitation

Baseline index	Model 1			Model 2		
	Odd ratios	95% CI	p value	Odd ratios	95% CI	p value
FEV_1_%predicted, %	1.02	0.97–1.08	0.35	1.06	1.00–1.13	0.046
6MWD, m	1.00	0.98–1.02	0.69	1.00	0.99–1.02	0.65
mBorg scale dyspnoea	1.15	0.62–2.15	0.66	1.04	0.58–1.87	0.90
PI_max_, cmH_2_O	†			1.08	1.02–1.15	0.013
DE_max_, mm	1.37	1.09–1.71	0.006		†	
QMS, kgf/kg^*100^	1.10	1.00–1.20	0.053	1.06	0.98–1.15	0.17
R^2^	0.53			0.44		

*FEV*_*1*_ forced expiratory volume in 1 s, *6MWD* 6-min walk distance, *mBorg* modified Borg, *PI*_*max*_ maximum inspiratory pressure, *DE*_*max*_ maximum diaphragmatic excursion, *QMS* quadriceps muscle strength. ^†^Variables not included in the model

## Discussion

This is the first study to demonstrate the utility of DE_max_ to predict the responsiveness of patients with COPD to 12-week PR. In this study, multivariate analysis revealed that greater baseline DE_max_ was the only factor that predicted the responsiveness to PR, independent of baseline %FEV_1_. Additionally, the model using DE_max_ had better prediction performance than that using PI_max_. The AUC of DE_max_ to predict the 30 m or more improvement in 6MWD after the PR was 0.915, with a sensitivity of 83% and a specificity of 95% at 44.9 mm.

PR is beneficial to patients with chronic respiratory disease, including COPD [3], and generally improves exercise performance, health-related quality of life and dyspnoea [22], which was confirmed in this study. Ideally, PR was proven to be effective in all patients, but the response to PR varies considerably between individual patients [8, 23–25]. Indeed, in this study, the improvement in 6MWD was less than that in MCID in 40% of the patients regardless of the degree of severity of COPD. Therefore, identifying predictors of a response is crucial in ensuring better PR efficacy and personalisation of PR programmes for patients with COPD.

In this study, the baseline values of %FEV_1_, PI_max_, DE_max_, QMS and 6MWD were positively associated with Δ6MWD in univariate analysis, suggesting that a better baseline condition was associated with a higher proportion of patients who achieved MCID after PR. These findings are consistent with those of previous studies that showed that patients with higher levels of %FEV_1_ or FEV_1_/VC achieved greater improvement in 6MWD after PR [7, 26, 27] and a study in which patients with milder mMRC scores could achieve MCID of 6MWD after PR [8], but not for those with worst mMRC score, although others studies showed contradictory results [5, 6, 28–30] or found no significant baseline characteristics to predict a response to PR [31]. The discrepancy between the findings cannot be fully explained, but it might be due to the differences in the studied population and strength or length of PR. In this study, the mean %FEV_1_ of the participants was 56.0%, which was relatively higher than that of other studies (mean %FEV_1_ of 40–50% in most studies) [5, 6, 28], despite similar inclusion criteria throughout the studies, i.e., not limited to severe COPD in most studies. Thus, no ceiling effect with a PR programme that included high-intensity load exercise training for 20–40 min was observed in our population.

In this study, an important finding is that greater DE_max_ at baseline was the only factor that predicted the responders in 6MWD after PR. In addition, the model using DE_max_ had better prediction performance than that using PI_max_. The high predictability of DE_max_ may be because of its strong association with DLH and dyspnoea during exercise, as reported previously [9]. DLH is involved in the development of dyspnoea, and both are important factors to determine the improvement in 6MWD in patients with COPD. Therefore, DE_max_ that reflects the degree of DLH and dyspnoea during exercise was superior to other physiological indices to predict responders.

Furthermore, the virtuous cycle observed in our PR programme that included high-intensity load exercise training might be a result of the improvement in ventilation pattern. Improving the ventilation pattern would be easier with greater DE_max_, as shown in studies of mechanically ventilated patients [32], which may have reduced dyspnoea during exercise after 12 weeks of PR and improved exercise tolerance. Exercise therapy is a central component of PR, which significantly reduces blood lactate levels during exercise, reduces minute ventilation and improves exercise tolerance [33]. The high-intensity load exercise training, which is performed at 60–80% of the maximum oxygen uptake, has a higher physiological effect than low exercise load. Patients with greater DE_max_ may be able to perform higher load training, which resulted in effective PR.

Diaphragm ultrasonography has been widely and successfully used to identify diaphragmatic dysfunction by showing its association with 6MWD, dyspnoea [10], extubation failure in mechanically ventilated patients [32], and increased mortality [11]. Recently, Lewinska and Shahnazzaryan proposed its use in pulmonary physiotherapy of patients with COPD [34]. In most previous studies, diaphragm ultrasonography was used to assess DE_max_, i.e., the measurement of the excursion of the right hemidiaphragm, as used in this study, and diaphragm thickness that assessed the length and thickness of the zone of apposition of the diaphragm against the rib cage [35, 36]. However, it is difficult to measure diaphragm thickness in patients with severe COPD because the length of the zone of apposition is shorter in patients with COPD than that in control subjects [37], whereas it is easy to measure DE_max,_ which shows high intra- and inter-observer reliability [38]. Bhatt et al. showed that improvement in 6MWD was associated with that in DE_max_ during forced expiration when the effectiveness of pursed lips breathing was assessed in the PR of patients with COPD [39]. Corbellini et al. demonstrated greater improvement in DE_max_ during inspiration after PR, which was associated with an increase in the inspiratory capacity [40]. The normal and cut-off values of DE_max_ during normal respiration, forced respiration, and voluntary sniffing have been described for each gender [38]. Thus, DE_max_ would be a useful and reliable measure for incorporation into the PR assessment. Furthermore, in clinical settings, this objective measure of DE_max_ has additional advantages as it requires minimum effort in patients and can be applied to the PR programme at home if portable ultrasonography is used. However, the assessment of DE_max_ has a limitation. The procedures pertaining to positioning of patients, breathing patterns, and the selected hemidiaphragm are not standardised at present, which may hamper the routine use of DE_max_ at this moment. Standardisation of these parameters would further facilitate the use of DE_max_ in clinical settings and for research purpose.

There are some limitations to this study. This was a single-centre study involving a relatively small number of participants, and their baseline condition might have been relatively preserved. Nonetheless, 46% of the participants showed FEV_1_ < 50%, and the utility of DE_max_ was also observed in these patients with severe airflow limitation. Furthermore, in this study, few patients discontinued the PR programme, except for patients who discontinued during the coronavirus pandemic, which indicates that there was no severe mismatch between the PR programme and the patients’ ability to successfully complete this programme. As another limitation, we did not evaluate any malnutrition factors, which could be an important determinant of diaphragmatic mobility. Nonetheless, DE_max_ was a stronger predictor of the effectiveness of PR than other parameters, including QMS or lung function using multivariate analysis. Further studies with a large number of patients are required, and the utility of DE_max_ should be examined in patients with the most severe form of COPD with a low-intensity load exercise programme.

## Conclusion

In conclusion, DE_max_, which is a reliable and easy to perform measurement, could adequately predict the improvement in exercise tolerance after PR in patients with COPD. Assessment of DE_max_ could aid in making medical decisions associated with therapeutic strategies.

## Data Availability

The datasets used and/or analysed during the current study are available from the corresponding author on reasonable request.

## Abbreviations

COPD: Chronic obstructive pulmonary disease
DLH: Dynamic lung hyperinflation
PR: Pulmonary rehabilitation
6MWD: 6-Min walk distance
MCID: Minimal clinically important difference
FVC: Forced vital capacity
FEV1: Forced expiratory volume in 1 s
PImax: Maximum inspiratory pressure
QMS: Quadriceps muscle strength
CAT: COPD assessment test
%LAA: Low attenuation area
AUC: Area under the curve
ROC: Receiver operating characteristic
mBorg: Modified Borg
